# Novel contribution of epigenetic changes to nuclear dynamics

**DOI:** 10.1080/19491034.2019.1580100

**Published:** 2019-03-17

**Authors:** Marcel Dreger, Elena Madrazo, Adam Hurlstone, Javier Redondo-Muñoz

**Affiliations:** a Faculty of Biology, Medicine and Health, Division of Cancer Studies, School of Medical Sciences, The University of Manchester, Manchester, UK; b Department of Immunology Ophthalmology and ENT, Hospital 12 de Octubre Health Research Institute (imas12), Complutense University, School of Medicine, Madrid, Spain; c Lydia Becker Institute for Inflammation and Immunity, The University of Manchester, Manchester, UK

**Keywords:** Epigenetics, mechanogenomics, chromatin, cell migration, nuclear mechanics

## Abstract

Migrating cells have to cross many physical barriers and confined in 3D environments. The surrounding environment promotes mechano- and biological signals that orchestrate cellular changes, such as cytoskeletal and adhesion rearrangements and proteolytic digestion. Recent studies provide new insights into how the nucleus must alter its shape, localization and mechanical properties in order to promote nuclear deformability, chromatin compaction and gene reprogramming. It is known that the chromatin structure contributes directly to genomic and non-genomic functions, such as gene transcription and the physical properties of the nucleus. Here, we appraise paradigms and novel insights regarding the functional role of chromatin during nuclear deformation. In so doing, we review how constraint and mechanical conditions influence the structure, localization and chromatin decompaction. Finally, we highlight the emerging roles of mechanogenomics and the molecular basis of nucleoskeletal components, which open unexplored territory to understand how cells regulate their chromatin and modify the nucleus.

The nucleus is the most complex and organized organelle within the cell. It comprises several components, including the nuclear envelope, the lamina network, chromatin and many other subnuclear structures [1]. The nuclear envelope partitions the genetic material from the cytoplasm and is a complex structure formed by a double lipid bilayer membrane (the outer and inner membranes), the nuclear pores and lamins (type A and B), which are intermediate filaments that assemble into the nuclear lamina. The nuclear lamina is connected to the inner nuclear membrane and the chromatin and plays fundamental roles as a nuclear scaffold, mechanosensor, genome regulator and in human pathologies [2]. Chromatin is composed of the genomic DNA and structural components such as histones. Global and local chromatin structure rearrangements are critical for many cell functions, including gene replication, repair, transcription and the cell cycle [3].

This Extra View article highlights the role of chromatin organization in nuclear deformation required for 3D cell migration. First, we will review recent studies that describe the influence of chromatin on the mechanical properties of the nucleus. Then, we will discuss how epigenetic changes are related with non-genomic functions and their importance during cell migration upon different stimuli. Finally, we will integrate our recent findings on how WDR5 activity and H3K4 methylation influence 3D cell migration, presenting novel evidence for the importance of the cyto- and nucleo-skeletal machinery on cell migration.

## Chromatin contribution to nuclear stiffness

Nuclear deformability and how the nucleus alters its morphology to allow cells to cross physical barriers and migrate through confined spaces has been extensively studied over the past 15 years [4,5]. First, lamin A/C expression was defined as a central contributor to nuclear morphology and deformability [6]. Likewise, cells surrounded by tissues with different mechanical properties present altered ratio of lamin A/C and lamin B, which indicated that nuclear lamin expression controls the mechanical properties of the nucleus in response to the microenvironment [7]. Although most research has been performed on lamins, now it is broadly accepted that chromatin configuration also contributes to nuclear rigidity [8–13].

The structure of chromatin is regulated by epigenetic changes, which are defined as non-genomic modifications that alter chromatin compaction and configuration [14]. Several seminal contributions show how the balance between open and condensed chromatin controls nuclear shape and volume in nuclear swelling experiments [15,16]. It is well known that the addition of divalent cations promotes a condensed chromatin state and increases nuclear stiffness, while treatment with epigenetic drugs opens the chromatin and softens the nucleus [15,16]. Recently, studies have demonstrated the contribution of the chromatin to nuclear rigidity and small deformations of the nucleus, whilst nuclear lamins and their connections with the nuclear envelope contribute mainly to larger deformations [11,12]. Likewise, it has been reported that the nucleosome disposition induced by histone tails and DNA linkers are crucial for nuclear rigidity [17,18]. In addition, others previously showed that chromatin condensation is associated with lower nuclear deformability in mesenchymal stem cells (MSCs), which reinforces the idea that chromatin controls a different biomechanical response than lamins [10,19]. It has been suggested that mechanoadaptation of the chromatin occurs faster than nuclear lamina alterations [13,20]. Aligning with these studies, we have previously described that epigenetic changes related with closed chromatin conformation alter the mechanical properties and shape of nuclei from lymphocytes and leukemia cells [21]. Now, we have extended these results and described how increased levels of a euchromatin marker (H3K4 methylation) are linked to the biomechanical properties of isolated nuclei from cells moving in 3D environments [22].

Usually, nuclear stiffness is studied in isolated nuclei or in intact cells. Both technical approaches have strengths and weaknesses: measurements in isolated nuclei are more precise whereas intact cells represent the more physiological environment. More than 10 years ago, several groups performed multiple biophysical approaches (including micropipette aspiration, atomic force microscopy, optical and magnetic tweezers etc.) to demonstrate that chromatin structure contributes, independently of its transcriptional activity, to the shape, size and mechanical properties that govern nuclear deformability [13,23,24]. Through atomic force microscopy (AFM) and nuclear multiparticle tracking (NMPT) we have recently revealed that nuclei from cells in suspension or from cells in 2D culture present different stiffness and viscosity than nuclei from cells moving in 3D [22]. Our findings suggest that chromatin decompaction induced by confined conditions decreases nuclear stiffness. As a complementary approach, to analyze how chromatin alterations are responsible for the biophysical response of the nucleus, we also performed nuclear swelling experiments, similar to those reported previously [16]. Consistent with our observations, 3D environments promote chromatin decompaction, which in turn contributes to the regulation of mechanical properties of the nucleus. Thus, the chromatin architecture is affected by mechanical signals in multiple conditions, including cell migration. Moreover, studies are beginning to establish a role for the chromatin structure that influences the physical properties of the nucleus but further analyses about the role of open or closed chromatin conformation must be performed in the future.

## Chromatin contribution to cell migration across confined spaces

Cell migration is a fundamental process critical for multiple physiological and pathological functions, including embryonic development, tissue remodeling, immune response and cancer [25]. Cells integrate the physical and biochemical properties of the tissues and interstitial spaces by promoting specific biomechanical responses in a process called mechanotransduction [26]. In general, the properties of the extracellular matrix (ECM), such as its stiffness or molecular architecture, promote specific physical and biochemical responses from moving cells resulting in cytoskeletal rearrangements, protease secretion and nuclear alterations [27]. Over the past fifteen years, a growing body of evidence has shown that these nuclear alterations include nuclear rotation and positioning, the movement of the nucleus (acting as a piston in lobopodial migration), and high nuclear deformation [28,29]. An ongoing research field is to understand how moving cells adapt their nuclei to the specific context of the microenvironments. A plausible hypothesis is that the balance between a stiff nucleus, which can apply forces and open gaps through endothelial barriers [30], and a soft nucleus, which is required for cell squeezing through extracellular spaces, might be critical for cell movement.

Experiments in melanocytes demonstrated that cell migration in 2D, which does not require nuclear deformability, leads to histone methylation in heterochromatin markers, such as methylation of H3 at residues K9 or K27 [31,32]. These chromatin changes might control the cell polarity, the migratory phenotype, and the mechanical properties at specific nuclear regions that might be linked to the cytoskeleton and a more efficient cell movement. This effect has been described in multiple cancer cells, which condense their chromatin during cell migration, affecting also their global chromatin conformation and DNAse sensitivity [31,32]. In other cases, the mechanical microenvironment promotes increased levels of euchromatin markers [33]. How the chromatin responds to mechanical forces has recently been defined as mechanogenomics [34,35]. This opens new questions about how mechanical stress might promote DNA damage and foster the generation of tumor mutations.

Another relevant aspect of the nuclear biology field is the formation of nuclear blebs. Nuclear blebs are nuclear protrusions observed during cell migration in confinement. These nuclear herniations also promote transient nuclear envelope ruptures during cancer cell migration that compromise the nuclear and genomic integrity. Interestingly, DNA repair enzymes localize at these places [36,37]. Moreover, these nuclear blebs are linked to chromatin alterations [12] and have fundamental roles during transendothelial migration of leukocytes [30]. We have described how G9a activity (a histone H3K9 methyltransferase) is also important for transendothelial migration of acute leukemia cells [38]. Our studies indicate that a dense collagen matrix promotes H3K4 methylation in leukocytes [22]. Furthermore, we could show that the inhibition or silencing of WDR5 (WD Repeat Domain 5), a core subunit of the histone H3K4 methyltransferase, diminishes the number of cells with highly deformable nuclei [22]. We have described new non-transcriptional functions for WDR5, which align with the idea that specific chromatin changes are uncoupled from gene activation [39]. Interestingly, cell migration through small pores leads to chromatin compaction and the exclusion of mobile nuclear proteins from the areas of nuclear rupture [40,41]. However, it has been reported that mechanical deformation promotes altered gene expression programmes [42,43]. Thus, we cannot entirely discard transcriptional consequences or nuclear bleb formation induced by WDR5, and future follow-up work will explore this question. It is widely accepted that chromatin regions are located in specific nuclear regions called chromosomal territories [44]. New studies support the idea that the disposition of these chromosomal territories is not random and dependent on external and internal stimuli, such as cell geometry and cell-matrix connections, which then promotes gene transcription, lamin anchorage and chromosomal stretching [45]. An ongoing question is how the chromatin structure contributes to nuclear polarity and deformability, thereby facilitating cell movement in confined spaces.

## Chroma-skeletal connections

The functional connections between the nucleus, membrane receptors and the cytoskeleton was pointed out more than 20 years ago [46]. Studies suggest a role for the cytoskeleton on mechanogenomics and gene regulation, recently reviewed in [47,48]. However, the precise molecular mechanisms of this regulation remain elusive. For example, it is known that actin polymerization controls the nuclear localization of the transcription factors YAP (Yorkie-homologues YAP, Yes-associated protein) and TAZ (transcriptional coactivator with PDZ-binding motif) [49]. Furthermore, the functional connections between the cytoskeleton (actin, tubulin and intermediate filaments) and the LINC (linker of nucleoskeleton and cytoskeleton) complex are required for nuclear mechanotransduction [23,50]. Our observations demonstrate that myosin contraction induced by constricted conditions controls H3K4 methylation induced by WDR5 [22]. It has been previously reported that cell confinement regulates actomyosin mechanics, which, in turn, control nuclear morphology as well as histone and telomere changes in constraint culture conditions [51]. Accordingly, we have seen that myosin is phosphorylated in constricted conditions, which stimulates H3K4 methylation [22].

Actin and actin-related proteins (ARPs), including actin partners such as fascin, control nuclear shape and chromatin remodeling [52]. Furthermore, WASP (Wiskott-Aldrich syndrome protein) and FAK (focal adhesion kinase) also localize in the nucleus and control epigenetic changes especially in leukocytes [53,54]. Two recent publications have demonstrated that nuclear actin and ARPs control heterochromatin integrity and DNA damage, highlighting the importance of DNA integrity for cell migration [55,56]. We have revealed that myosin phosphorylation *via* MLCK (myosin light chain kinase) activity is required for WDR5 function. Moreover, MLCK localizes partially at the euchromatin areas of the nucleus [22]. Although we cannot yet determine which MLCK localization is essential for the epigenetic regulation induced by 3D constricted conditions, the results demonstrate that MLCK, besides its cytoplasmic localization, also localizes in the nucleus of lymphocytes. However, how mechanical signals control the nuclear import of cytoskeletal components or their activation in the nucleus is poorly understood. Unraveling these dynamic processes will be crucial to gain fundamental knowledge about the mechanical and molecular pathways that influence nuclear alterations in response to constricted conditions.

## Summary and conclusions

Nuclear deformability and its importance for cell migration in confined spaces is gaining constant interest in the scientific community. Current research has shown that the mechanical properties of the ECM modulate nuclear mechanics. We have introduced several recent studies that show how mechanogenomics comprise chromatin changes induced by mechanical properties of the surrounding microenvironment. Chromatin configuration not only acts as a transcriptional platform, but also contributes to the nuclear response to external stimuli, thereby promoting effective cell migration. Interesting future studies include how epigenetic changes are linked to DNA damage and if epigenetic changes are sufficient to diminish DNA damage upon nuclear deformation. Another area of interest is to understand the long-term effects of epigenetic changes and histone modifications on transcriptional programmes. We have shown that mechanical changes of the nucleus seem to be linked to the actomyosin contractility. However, the role of cytoskeletal (or nucleoskeletal components) during epigenetic alterations is still missing, and represents a major challenge for the future. Unraveling the mechanism between the cytoskeleton and the chromatin structure will improve our understanding of the epigenetic machinery and nuclear deformability
